# Automated cornea diagnosis using deep convolutional neural networks based on cornea topography maps

**DOI:** 10.1038/s41598-023-33793-w

**Published:** 2023-04-21

**Authors:** Benjamin Fassbind, Achim Langenbucher, Andreas Streich

**Affiliations:** 1 Department of Computer Science, Lucerne University of Applied Sciences and Arts, Rotkreuz/Zug, 6343 Switzerland; 2 Department of Experimental Ophthalmology, Saarland University, Homburg/Saar, 66123 Germany

**Keywords:** Preclinical research, Corneal diseases

## Abstract

Cornea topography maps allow ophthalmologists to screen and diagnose cornea pathologies. We aim to automatically identify any cornea abnormalities based on such cornea topography maps, with focus on diagnosing keratoconus. To do so, we represent the OCT scans as images and apply Convolutional Neural Networks (CNNs) for the automatic analysis. The model is based on a state-of-the-art *ConvNeXt* CNN architecture with weights fine-tuned for the given specific application using the cornea scans dataset. A set of 1940 consecutive screening scans from the Saarland University Hospital Clinic for Ophthalmology was annotated and used for model training and validation. All scans were recorded with a CASIA2 anterior segment Optical Coherence Tomography (OCT) scanner. The proposed model achieves a sensitivity of 98.46% and a specificity of 91.96% when distinguishing between *healthy* and *pathological* corneas. Our approach enables the screening of cornea pathologies and the classification of common pathologies like *keratoconus*. Furthermore, the approach is independent of the topography scanner and enables the visualization of those scan regions which drive the model’s decisions.

## Introduction

Vision is arguably the most important way we humans navigate and interact with our environment. However, we tend to only notice this once the visual performance is deteriorated due to some pathology. Keratoconus, for example, is a progressive eye disease that affects the cornea of the eye, and is one of the most common cornea pathologies^1^. The cornea reduces in thickness, and thus starts to bulge out due to the intraocular pressure, an effect that typically results in visual deterioration^2,3^. In advanced stages, a cornea transplant is the only feasible treatment option. If diagnosed early, however, less invasive treatments like Corneal Cross Linking (CXL) or Intracorneal Ring Segments (ICRS) can be used to reduce disease progression^4^.

When visiting an ophthalmologist, often the corneal topography is inspected using an OCT scan. OCT scans are non-invasive and are often part of standard diagnostic procedures in ophthalmology. However, especially in early stages, keratoconus and other corneal pathologies are hard to detect, making a diagnosis based on these scans extremely difficult. According to a study from the Department of Opthalmology at the Cantonal Hospital of Lucerne^5^, the difficulty of diagnosis may also be compounded by weaker than expected levels of *keratoconus* expertise among general ophthalmologists. Some corneal topography devices already calculate an index like the CASIA2 Ectasia Severity Index (ESI)^6^ or the Pentacam Belin-Ambrósio enhanced ectasia display (BAD)^7^, on how deformed a cornea is. Some assumptions underlying those indexes, like the separation of the cornea in < 3 mm and > 3 mm zones in the BAD or the focus on asymmetry in the ESI (thus neglecting central *keratoconus* cases), seem arbitrary to the authors. Those algorithms are also unable to distinguish between different corneal pathologies^8^.

Data and image classification techniques have become increasingly relevant and useful in everyday life situations and also in the medical field^9–11^. Such techniques are successfully used in oncology^12^, dermatology^13^ and many other domains^14^ to help diagnose diseases.

Major advancements in the field of computer vision and image classification helped to improve the accuracy and reliability in such classification tasks^15^. Among such advancements, Convolutional Neural Networks (CNN) are a class of deep neural networks which take inspiration from the way mammal brains interpret vision signals from the eye^16^. CNN are especially powerful in analyzing unstructured data such as images. Using a deep learning approach, pathologies can be detected and classified automatically, without the need for experts to interpret indexes that are different for every device.

The main aim of this study was to develop a method of using preprocessed corneal OCT screening data to automatically assess corneal health, and potentially to discriminate between different diseases and conditions such as *keratoconus*. This could be of significant benefit in a screening setting as part of a standard OCT diagnosis, where identifying corneal pathologies and abnormalities at an early stage would allow appropriate treatments to be applied promptly, improving outcomes.

Using preprocessed OCT data from a CASIA2 device, we demonstrate a model initially developed for determining whether a cornea is healthy or abnormal, and evaluate the potential of further training the model to distinguish between individual disorders and abnormalities using more detailed labelled data.

## Methods

Our proposed process first represents the numerical results of OCT scans as images, and then use a CNN to diagnose corneal abnormalities, especially *keratoconus*. The visual representation of the OCT as basis for diagnosis has several advantages. Firstly it corresponds to the way humans identify abnormalities in the cornea; secondly, as the parts of the image that are most influential for a particular outcome of a neural network can be easily identified and visualized, results can be examined and checked (explainable AI, or XAI^17^); and finally, a visual representation is relatively independent of the device and the setting used.

As a first step, classical Machine Learning (ML) methods such as *k-means* and *random forest* have been trained and evaluated on the raw scan data as baseline. The use of raw scan data means that in the future other cornea diagnostics devices like corneal tomography or Scheimpflug devices^18^ may not be supported by these methods. All the devices however can produce topography maps as those maps are used for diagnosis by ophthalmologists. Also, these classical ML methods lag in performance compared to modern CNN architectures, and they were not considered further.

In terms of technology, the entire processing was done in the programming language *Python*, version 3.10, running on a computer with an *NVIDIA RTX A5000* GPU.

### Cornea scans dataset

All anterior OCT scans were performed with a CASIA2 anterior OCT device by the Saarland University Hospital Clinic for Ophthalmology as part of a standard patient screening procedure. The device calculates various features from the measured cornea data. For this study the *anterior axial refraction*, *posterior axial refraction*, *anterior elevation*, *posterior elevation* and *pachymetry* were used. The data is provided as a 2D matrix of (32 $\times$ 400) data points in a polar format. Some faulty scans by the device with missing or invalid data points were filtered out as part of a data quality assessment^19^. The CASIA2 masks data points where no valid data is available, for example when the eyelid obstructs the cornea. Scans with any masked value in the central 2mm area were discarded. This resulted in approximately 10% of the scans being filtered. Some of these scans for example had a *pachymetry* of several centimeters and more. Supplementary Figure S1 shows an example of a faulty scan.

The raw measurement data were represented as images using the function *contourf* in the Python package *matplotlib*^20^; these images were annotated by a cornea expert to obtain the labels for the supervised learning task. To support the annotation process, the keratoconus ABCD score^21^ and the asymmetry and astigmatism in the inner 1.5 mm of the cornea were also provided.

As each cornea scan could potentially contain any cornea pathology, the most common cornea pathologies and abnormalities were annotated. All technically well-recorded scans were used to get a realistic real-world data sample. Besides *keratoconus* (the most frequent pathologic condition), several other clearly defined conditions have been used as labels despite insufficient training data for a few of them. Specifically, the following labels were given: *healthy*, *keratoconus*, *post laser*, *keratoglobus*, *pellucid marginal corneal degeneration (PMD)*, *other* (less common diseases/scars/irregularities in the cornea) and *not appreciable (N/A)* (faulty, invalid or unidentifiable scans by the OCT device). Supplementary Figures S2 to S8 show examples of the different labels.

Traditional statistical approaches are not able to filter out all invalid scans. Some scans might look valid from a data distribution perspective but do not allow an ophthalmologist to assess the health condition. Those scans were labeled as *N/A* and then also used during model training, as in a clinical setting such invalid scans might be present.

The annotated 1940 cornea scans from 899 patients were randomly split into a *training* (containing *80%* of the available data) and *validation* dataset. Hold-out validation was performed to evaluate the models’ performance while training. Table 1 presents the split of the cornea scans dataset and the number of scans used for every set.

To evaluate the final model, a completely new, disjoint and more recent test dataset was collected, consisting of 242 scans from 188 patients. The scans were performed with the same CASIA2 device in the same clinic as the training and validation scans. The test dataset only contains scans from patients not present in the training or validation datasets. The labeling was done in the same way as the labeling of the training and validation dataset, but this time by two different cornea experts. For 15 (*6.20%*) scans the two experts disagreed on the label. These cases were discussed between the experts in order to agree on a final label.

**Table 1:** Class distribution and count in the cornea scans dataset showing the imbalanced classes in the screening data.

	Total	Healthy	Keratoconus	PMD	Post laser	Keratoglobus	Other	N/A
Train set	1552	719 (46.2%)	290 (18.6%)	8 (0.5%)	292 (18.8%)	4 (0.3%)	201 (12.9%)	38 (2.4%)
Val set	388	185 (47.6%)	61 (15.7%)	0 (0.0%)	88 (22.6%)	3 (0.8%)	44 (11.3%)	7 (1.8%)
Test set	242	113 (46.7%)	112 (46.6%)	0 (0.0%)	3 (1.2%)	2 (0.8%)	12 (5.0%)	0 (0.0%)

### Deep neural network architecture

Recent trends in computer vision using deep learning have shown that the *ConvNeXt* convolutional neural network architecture^22^ is well suited for classification of image data. Compared to traditional machine learning approaches, these architectures do not require domain knowledge to do feature engineering, as the relevant features are extracted automatically by the hidden layers of the neural network based on the training data. For this study, we have started with the existing *ConvNeXt* architecture provided by the Python package *torchvision*^23^ within the *pytorch* framework^24^.

**Figure 1:**
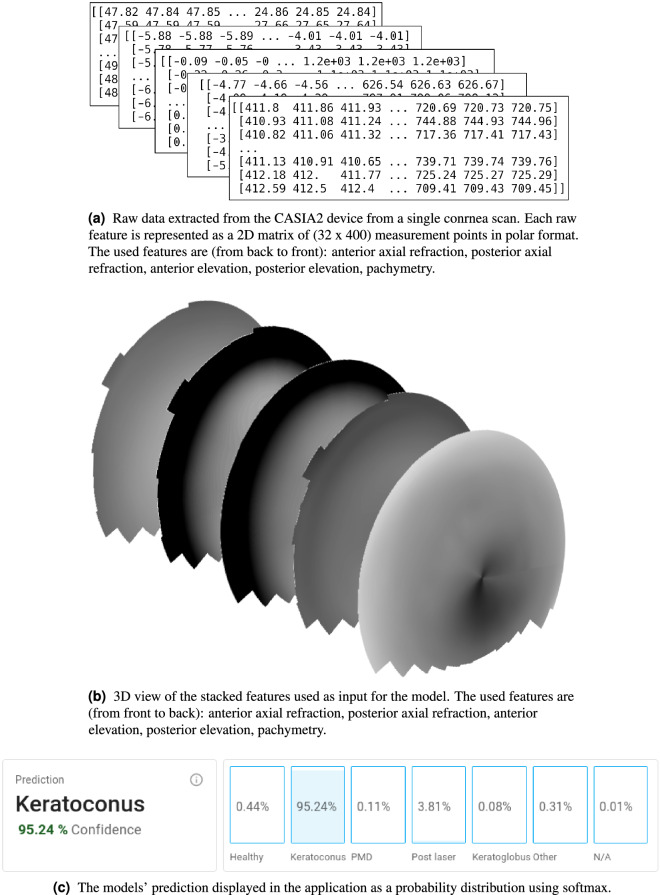
List of all the feature maps and how they are used as input for the *CorNeXt* model architecture. The topography maps are generated using *matplotlib*^20^ based on the exported raw data from the CASIA2 device. (**a**) The raw data extracted from the CASIA2 device from a single cornea scan. (**b**) The features anterior and posterior axial refractive and elevation and pachymetry stacked as the five input channels for the *CorNeXt* model. (**c**) The *softmax* probability distribution of the models’ prediction over all classes.

In order to use *ConvNeXt* for cornea disease classification, some adjustments to its architecture were necessary. For each cornea, the axial refractive power and the elevation of both the anterior and posterior surface of the cornea, as well as its thickness (pachymetry), are obtained from the scan. This results in five partially dependent maps, each of which can be represented as gray-scale image. Cornea abnormalities are then visible at the same location in several of these gray-scale images. As CNN architectures are powerful extractors of relevant features from spacial data, all gray-scale images of a cornea were stacked together to form a five-channel pseudo-image. The original *ConvNeXt* architecture takes three channels as input, one channel each for red, green and blue. For each of the three color channels, the information can be represented as gray-scale map. To make all available information from a cornea scan available to the model, the three channels from *ConvNeXt* are expanded to five channels, copying the pre-trained weights from *torchvision*. Figure 1 illustrates how the model receives the cornea maps as input, and how the predicted output is visualized. We call this new architecture *CorNeXt*.

The *CorNeXt* model was trained in multiple iterations to evaluate several model configurations. The final model hyper-parameter configuration is shown in Table 2. We found that training all weights of the CNN model resulted in better performance than just training the classification head. As a loss function negative log likelihood was used.

All weights of the model are transfer learned from the pre-trained *ConvNeXt* weights provided by *torchvision*. As optimizer *AdamW* is used, inspired by the original *ConvNeXt* implementation together with the *ReduceLROnPlateau* learning rate scheduler. While training, the model is augmented using *RandomVerticalFlip*. Augmentation is limited to *RandomVerticalFlip* because the position of the eye is fixed when creating an OCT scan.

To evaluate the model performance, the *accuracy*, *F1 score*, *sensitivity*, *specificity* and the *Area Under the Receiver Operating Characteristic (AUROC)* are calculated using the *torchmetrics* library^25^. The best model was selected based on the *F1 score* with a weighted average considering each class’s support. The *F1 score* is a method to measure a statistical test’s accuracy and allows a meaningful evaluation even when the dataset has an uneven class distribution.

**Table 2:** Hyperparameters of the final *CorNeXt* model.

Training dataset	Iterations	Loss	Optimizer	lr scheduler	Augmentation	Training time
Cornea scans dataset	35 k	Negative log likelihood	*AdamW* weight decay = 0.05 learning rate = $1e-6$	*ReduceLROnPlateau* monitor = val_loss mode = min patience = 30 factor = 0.1	*RandomVerticalFlip* p = 0.5	36 h

Determining whether a prediction was made for the correct reasons is the core topic of model explainability^17^. To do so, integrated gradient^26^ from the *captum*^27^ library was used to visualize which input features are most relevant for a model to make a certain prediction.

A *Python* based web application was developed to enable easy interaction with the *CorNeXt* model. In addition to that, the application allows researchers and ophthalmologists to visualize the data used for the prediction to verify whether the predictions are plausible.

## Results

**Figure 2:**
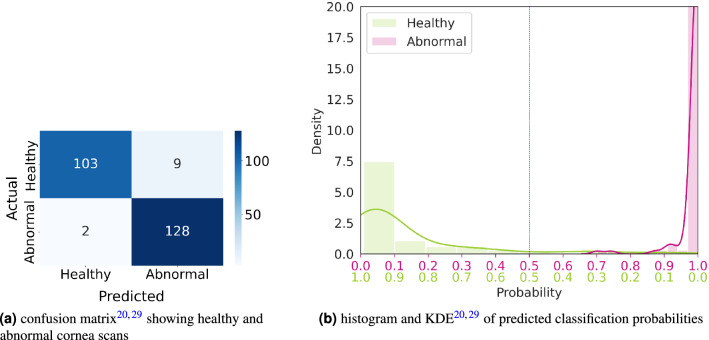
The performance of the *CorNeXt* model on the test cornea scans dataset in distinguishing between *healthy* and *abnormal* cornea scans. (**a**) The confusion matrix of the model. (**b**) How confident the model is when classifying a cornea scan as *healthy* or *abnormal*. The threshold for probabilistic model output was set to 0.5 (marked by the vertical line).

In a screening setting, the focus is on reliably identifying patients with any kind of cornea abnormality. For this evaluation, all metrics are evaluated as a binary classification consisting of the two classes *healthy* and *abnormal*. A scan is considered *abnormal* when it has been labeled as either *keratoconus*, *post laser*, *PMD*, *keratoglobus* or *other*. Note that all data points annotated as *n/a* were removed for this evaluation as it is not clear if the cornea is healthy or not in those cases. *Post laser* was treated as *abnormal* for two reasons: (1) namely before laser or cataract surgeries, it is essential to identify any cornea abnormality (including previous laser treatment), and (2) more often than not, *post laser* is misinterpreted as some other cornea pathology, since laser treatment can alter the shape of the cornea by quite some extent depending on the desired correction of the refractive error^28^. The evaluation result on the test cornea scans dataset is shown in Table 3. Figure 2 shows the confusion matrix and the confidence of the model when classifying a cornea as *healthy* or *abnormal* with respect to the correct label. A threshold of 0.5 on the softmax output was used to determine the predicted class. The *CorNeXt* model is able to distinguish between *healthy* and *abnormal* with very good performance.

**Table 3:** Evaluation results of the final model on the test cornea scans dataset when distinguishing only between *healthy* and *abnormal* cornea scans.

Metric	*Healthy* vs. *abnormal*
Accuracy	95.45%
F1 score	95.88
Sensitivity	98.46%
Specificity	91.96%
AUROC	0.9953

*Post laser* is treated as *abnormal* in this case.

In a further experiment, we investigate whether the model is able to yield a more granular interpretation of the scans and distinguish between different conditions. In general, the model identifies the different classes with varying performance. For all classes except *healthy* and *keratoconus*, there are too few samples in the test dataset to make reliable statements on model performance.

**Figure 3:**
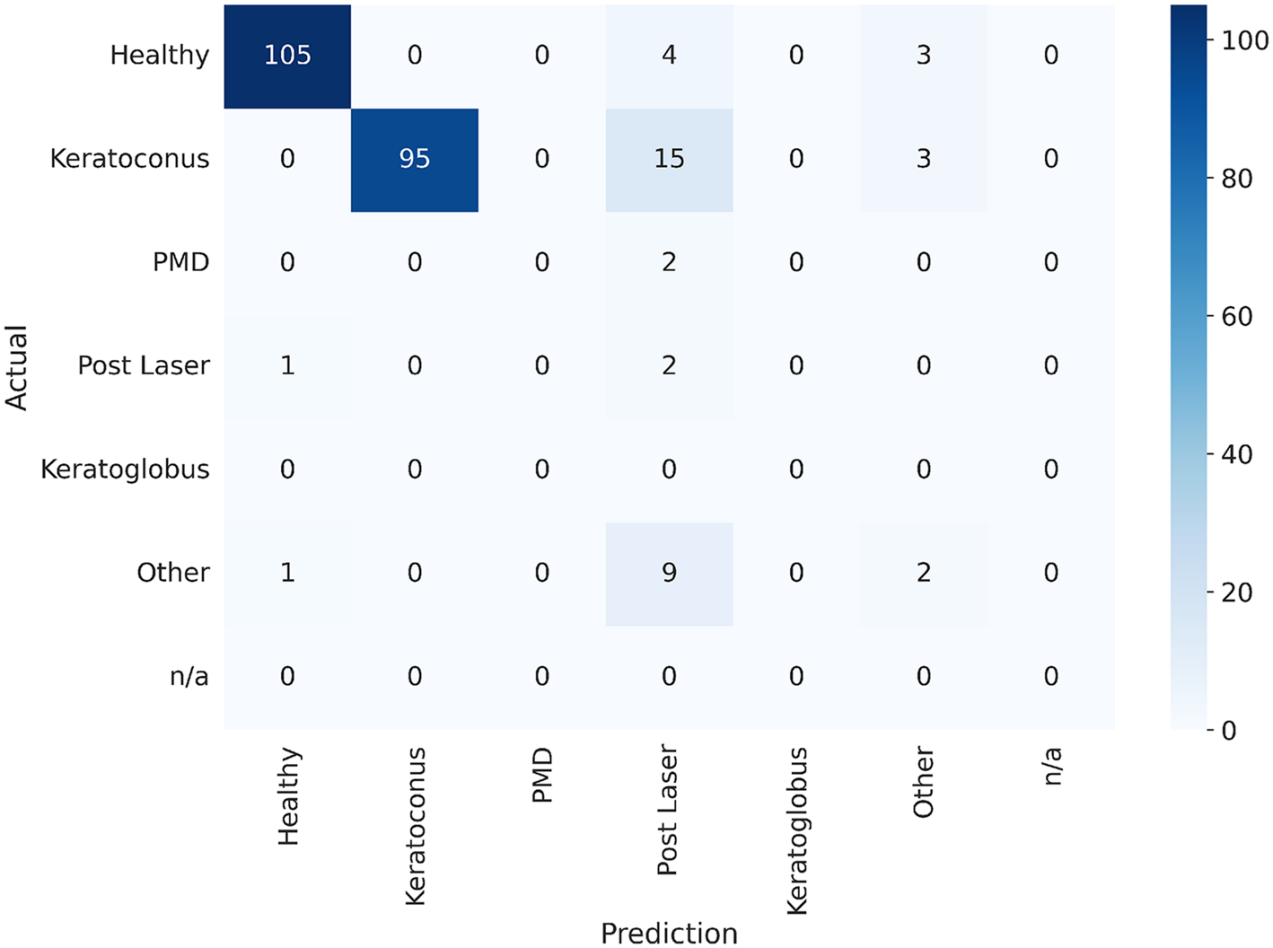
Confusion matrix^20,29^ of the test cornea scans dataset. The *Prediction* axis shows the actual prediction from the *CorNeXt* model and the *Actual* axis shows the ground truth labels.

The performance of the model on the test dataset is shown in Table 4 and the confusion matrix is displayed in Fig. 3.

**Table 4:** Evaluation results of the final model on the test cornea scans dataset.

Metric	Weighted average	*Healthy*	*Keratoconus*	*PMD*	*Post laser*	*Other*
Accuracy	93.52%	96.28%	92.56%	99.17%	87.19%	93.39%
F1 score	88.17	95.89	91.34	0.00	11.42	20.00
Sensitivity	84.30%	93.75%	84.07%	0.00%	66.67%	16.66%
Specificity	99.00%	98.46%	100.00%	100.00%	87.44%	97.39%
AUROC	0.9651	0.9952	0.9495	0.8604	0.8480	0.8771

Because the dataset is highly imbalanced, the average is weighted taking into account the support of each class. For the classes *n/a* and *keratoglobus* there are no samples in the test dataset, so they are omitted.

The performance of the model on the validation cornea scans dataset is reported in the supplementary material (Supplementary Tables S1, S2; Supplementary Figure S9). Comparing the performance on the different datasets can reveal potential overfitting: the comparison between validation and test data shows that the conclusions on the model architecture that were based on the performance of different variants on the validation set, are confirmed on new data.

When interpreting the results, it should be kept in mind that all the data is consecutive screening data. Hence, there might be a potential drift in the characteristics of the scanned eyes.

**Figure 4:**
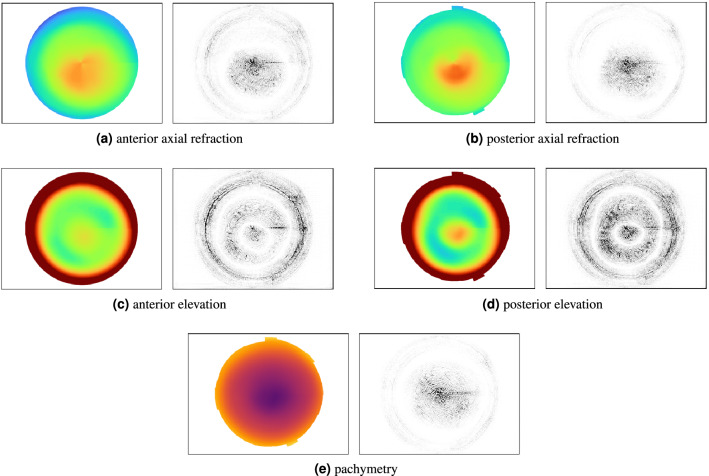
The computed integrated gradients with respect to the correct prediction *keratoconus* (88.9% confidence). A pixel highlighted in black means, that this pixel was relevant for the prediction process.

As the correct interpretation of an OCT scan is critial, we emphasize the importance of explainable artifical intelligence^17^. For image inputs, integrated gradients^26^ show which areas were most relevant to yield the predicted class label, and thus allow an expert to compare the key regions for the model with the most relevant area for a human-made diagnosis.

Figure 4 shows the integrated gradients on a correctly identified sample input scan with *keratoconus*. For each of the five input channels, a corresponding gray-scale heatmap depicts the importance of a given pixel in the input channel for the final label. The obtained class label is mainly based on the ectatic area in the cornea; which corresponds to the area deemed most important for a *keratoconus* by human experts.

**Figure 5:**
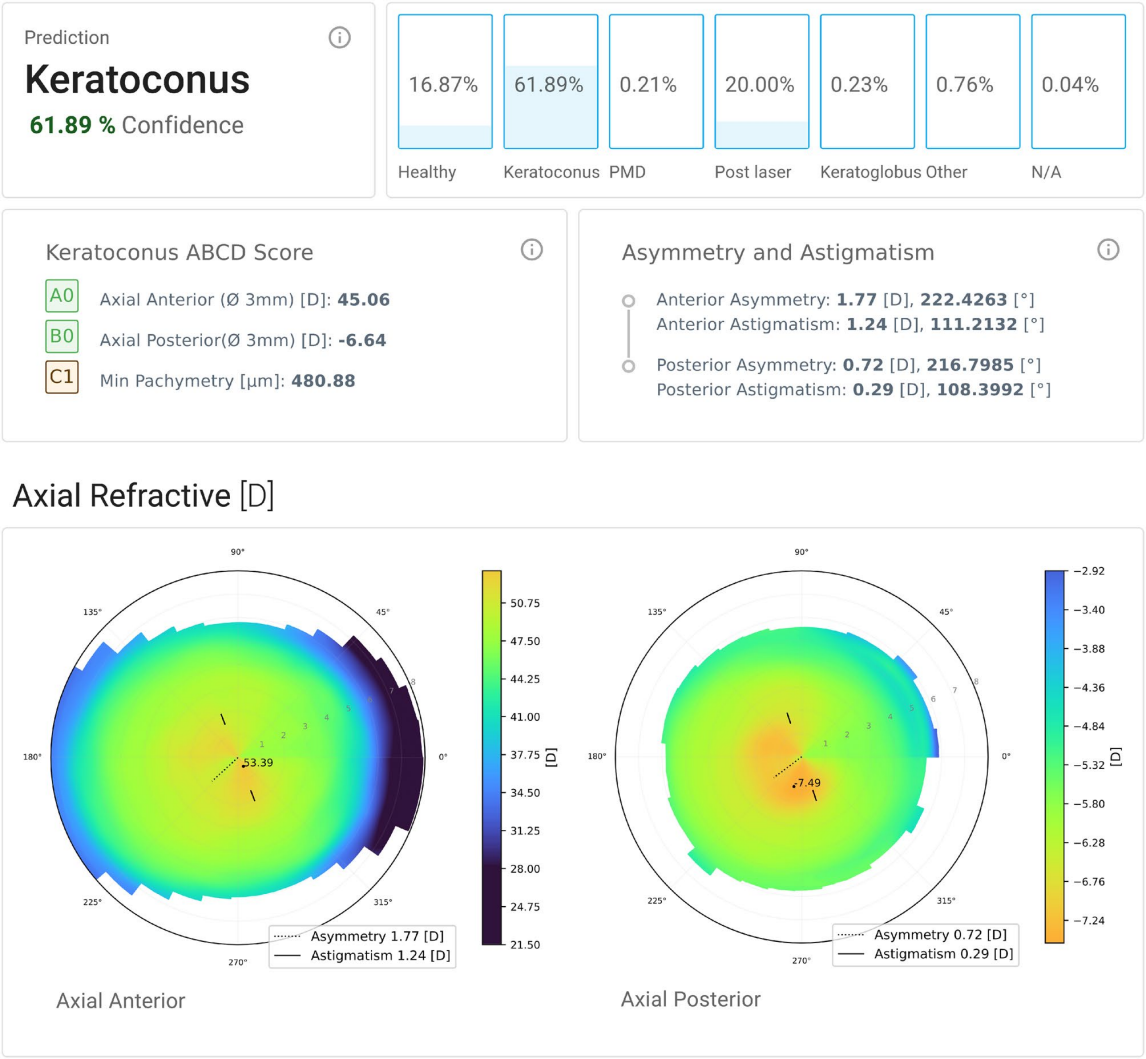
The scan details page of the developed Eye Analyzer application. At the very top the predictions of the *CorNeXt* model are visualized. This includes a probability distribution over all classes using a *softmax* output. Furthermore, the keratoconus ABCD score and asymmetry/astigmatism in the inner 1.5 mm region are displayed. The topography maps are also shown as a way to cross-check the models’ prediction results. Those maps are generated using the same methods as the input images for the model.

We also present a modern web application as shown in Fig. 5 to analyze anterior segment OCT scans and predict cornea pathologies. The application shows the probability for each of the most common cornea pathologies, predicted using the developed model. In addition to that, axial refraction, elevation and pachymetry maps and state-of-the art metrics like keratoconus ABCD score^21^ as well as asymmetry and astigmatism calculations are displayed to cross-check the prediction results.

## Discussion

The model accurately distinguishes healthy corneas from corneas with abnormalities. The classification of different types of abnormality heavily depends on the available training data and variance inside a class.

The performance between classes differs significantly. While *healthy* and *keratoconus* are well recognized, *post laser*, *keratoglobus*, *PMD*, *other* and *N/A* seem to be very challenging for the model. Unfortunately the test dataset does not contain enough samples of these classes to draw any meaningful conclusions about the performance of the model.

The confusion matrix in Fig. 3 shows that the model has difficulties distinguishing between the *post laser*, *keratoconus* and *healthy* classes. Looking at some samples of misclassification, we see that (1) the class *post laser* has quite a high variance and (2) some *post laser* treatments look very similar to *healthy* eyes. Hyperopic *post laser* treatments can also be misclassified as *keratoconus*, as the topography looks very similar to a *keratoconus* eye.

A comparison of the performance of the proposed *CorNeXt* with methods and results published elsewhere is challenging, mostly because there is no reference dataset. Similar works^30^ show large variations in terms of the types of cornea scanners used, sample size, validation methods and performance measurements. As far as we know, this study is the first to use a completely random sample set of consecutive screening data. Consequently, we believe that the results presented here come close to the performance that could be expected in a clinical setting.

An intuitive application called *Eye-Analyzer* was developed to enable ophthalmologists and researchers to easily analyze cornea OCT scans and interact with the AI model. The application can help to diagnose ectatic cornea diseases like keratoconus even in an early stage, where minimal invasive treatment options such as CXL or ICRS implementations are available. This might delay or even prevent corneal grafting or living with sometimes significantly decreased vision. The *Eye-Analyzer* application could also be used to analyze corneas before a cataract or laser surgery, looking for any abnormalities.

Currently, the *Eye-Analyzer* application can only be used for research purposes, as validation or certification as a medical product would be required to be used by ophthalmologists to diagnose patients corneas. Furthermore, some classes have barely satisfactory performance and more training data would need to be used to have a more stable performance predicting *keratoglobus*, *PMD*, *post laser* and *other*.

This study demonstrates that it’s possible to analyze anterior OCT cornea screening scans using CNNs. The proposed *CorNeXt* model is able to automatically assess corneal health with state-of-the-art performance compared to publicly available work^30^. Predicting the most common corneal diseases in screening data seems to be a much more challenging task. The performance of different classes varies depending on their variance and the number of samples available for training and validation. For some classes like *keratoglobus*, *PMD*, *post laser* and *other* there was insufficient validation data to draw any conclusions on their performance. Nevertheless, we still think it is important to not exclude this data, as it would artificially alter the dataset and could not be considered screening data anymore.

It is highly recommended to continue development of the model. The model predictions should also be compared to that of senior ophthalmologists specialized in cornea diseases. In addition to that, the dataset should be independently labeled by another ophthalmologist to better understand labeling differences between human experts. Using more training data would certainly also help to increase the model’s performance. This could be done either by labeling more data or adding an application feature for users to give feedback on the prediction results. Furthermore, a gold standard challenge to diagnose cornea abnormalities could be created, making comparison of such methods more reliable in the future.

## Supplementary Information

Supplementary Information.

## Data Availability

The data that supports the findings of this study was provided by Saarland University Hospital Clinic for Ophthalmology under license. The data can be made available upon request to the authors with permission of the Saarland University Hospital Clinic for Ophthalmology.
