# Supplementary material for: Automated cornea diagnosis using deep convolutional neural networks based on cornea topography maps
Source: Sci Rep. 2023 Apr 21;13:6566. doi: 10.1038/s41598-023-33793-w (PMC10121572; doi:10.1038/s41598-023-33793-w)
Supplement: Supplementary file 1 — Supplementary Information. [file 41598_2023_33793_MOESM1_ESM.pdf]

Automated Cornea Diagnosis using Deep Convolutional Neural Networks based on Cornea Topography Maps - Supplementary Materials

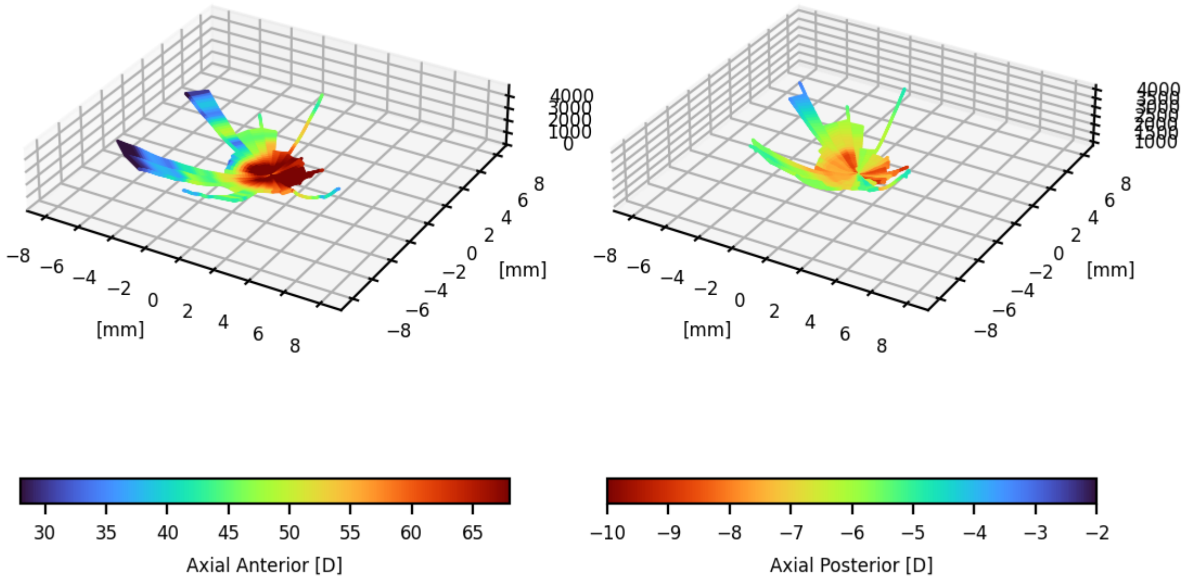

Figure S1. Cornea scan with missing data in the center 2mm area of the cornea.

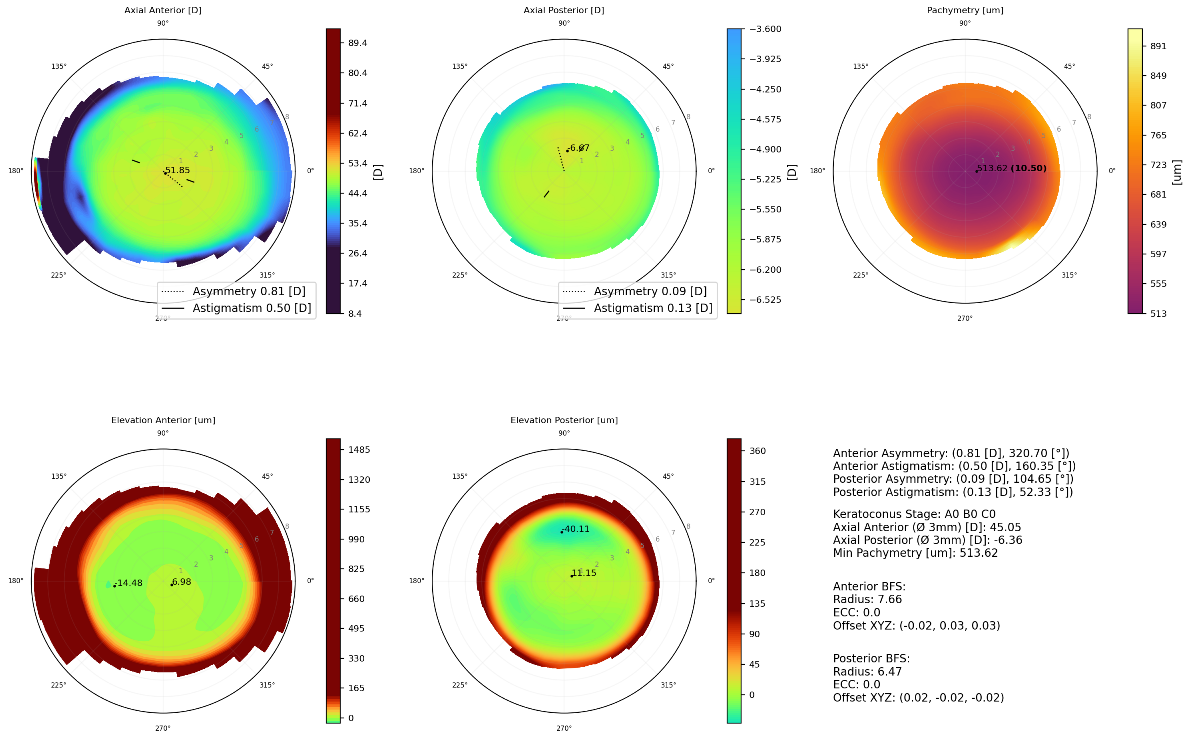

Figure S2. Scan labeled as "Healthy".

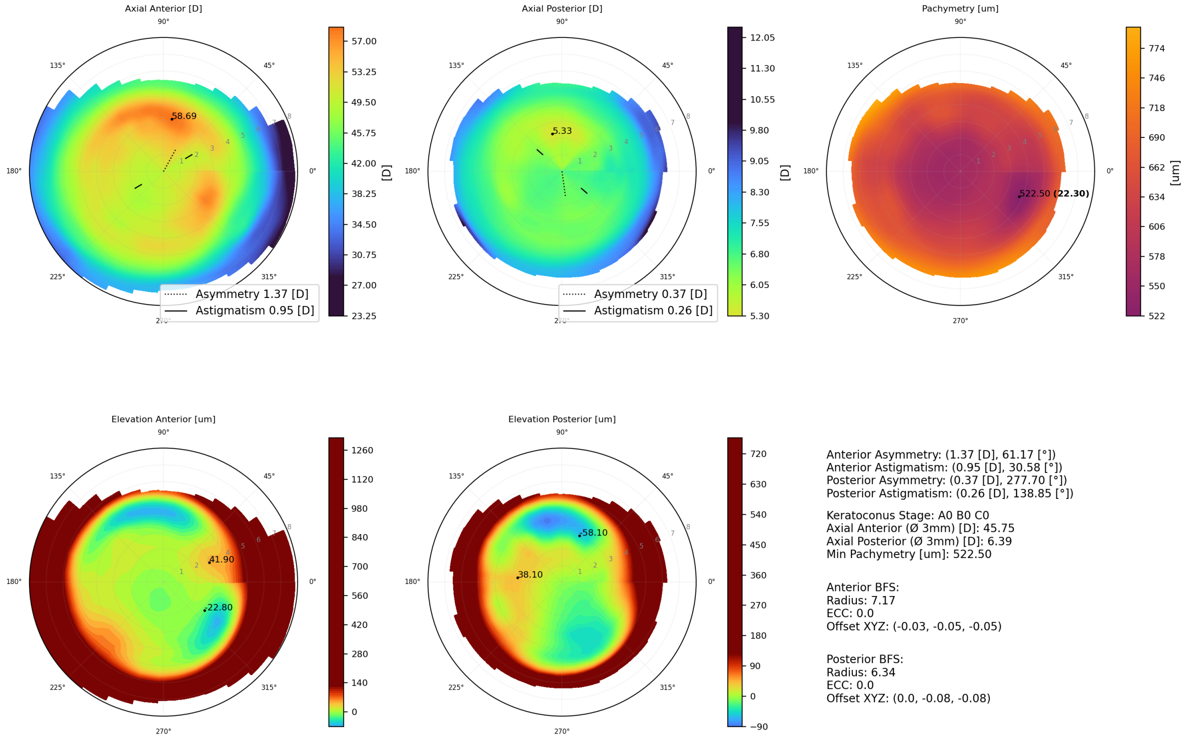

Figure S3. Scan labeled as "Post Laser".

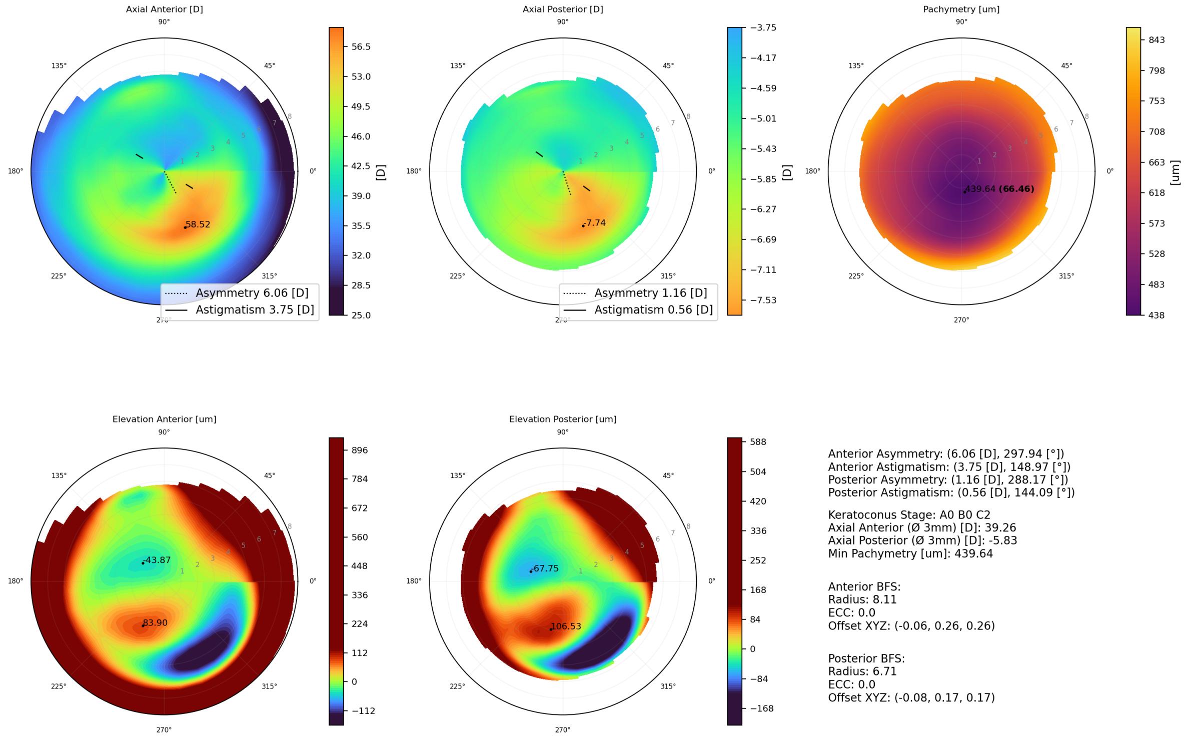

Figure S4. Scan labeled as "Keratoglobus".

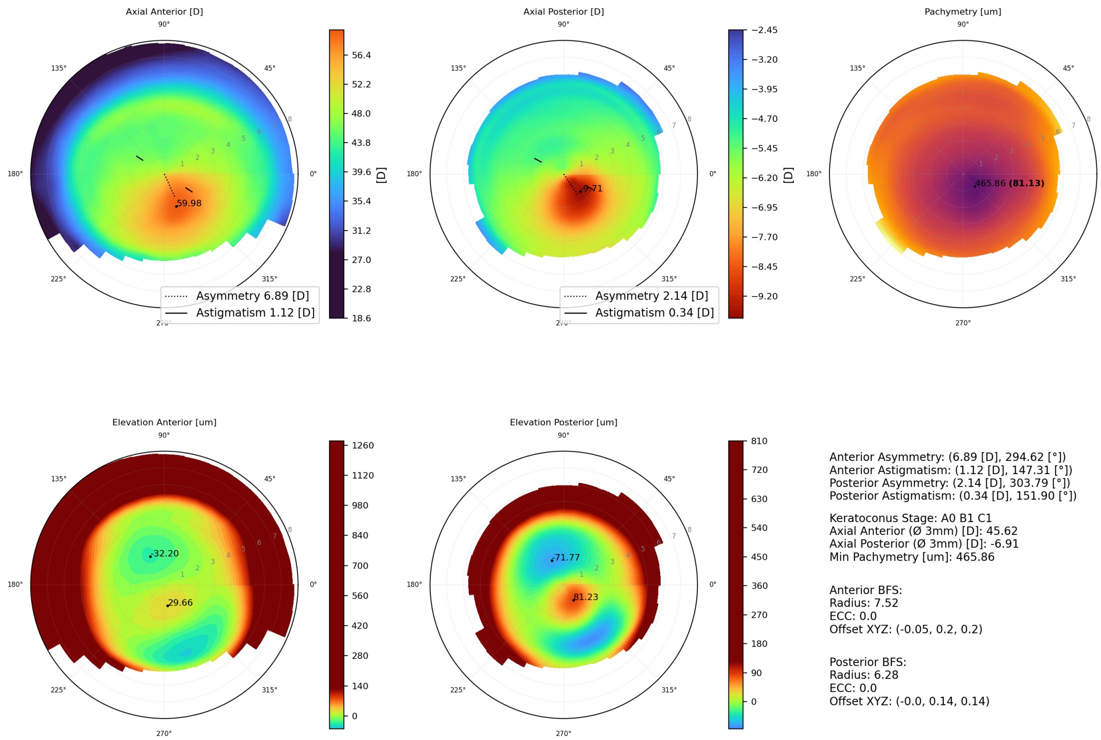

Figure S5. Scan labeled as "Keratoconus". Shows a clearly visible ectasia in the lower temporal quadrant of the eye.

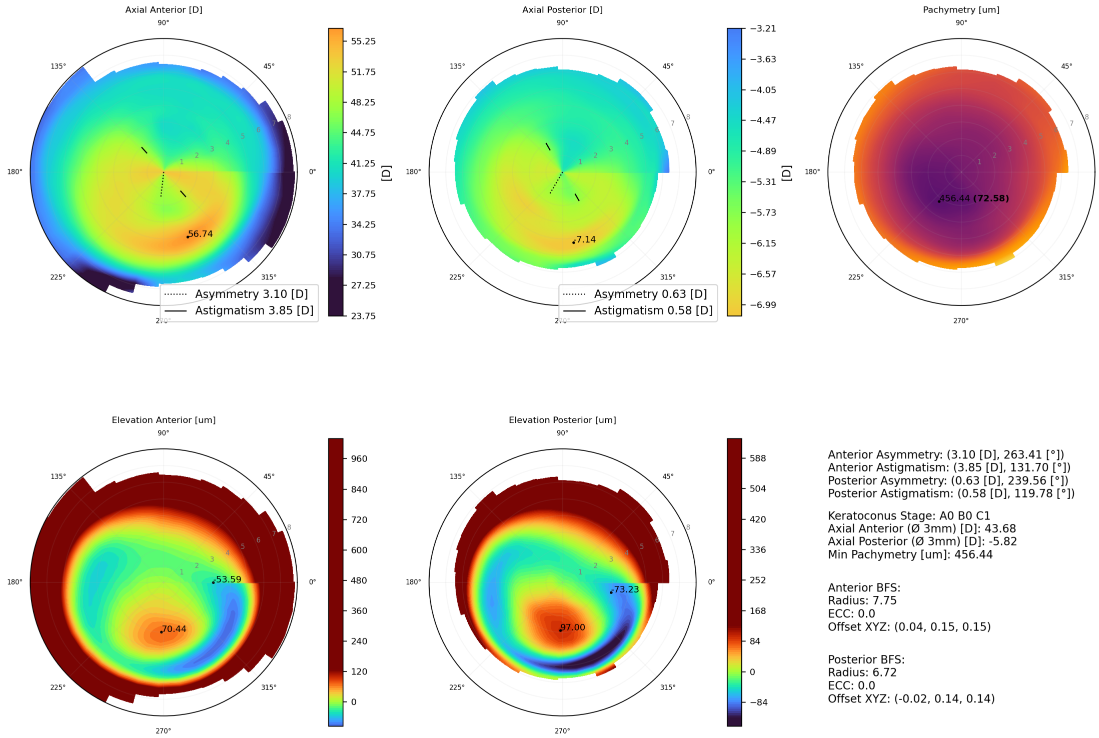

Figure S6. Scan labeled as "Pellucid marginal degeneration". Typical for this type of disease is the "kissing birds" pattern visible in the anterior and posterior axial refractive map.

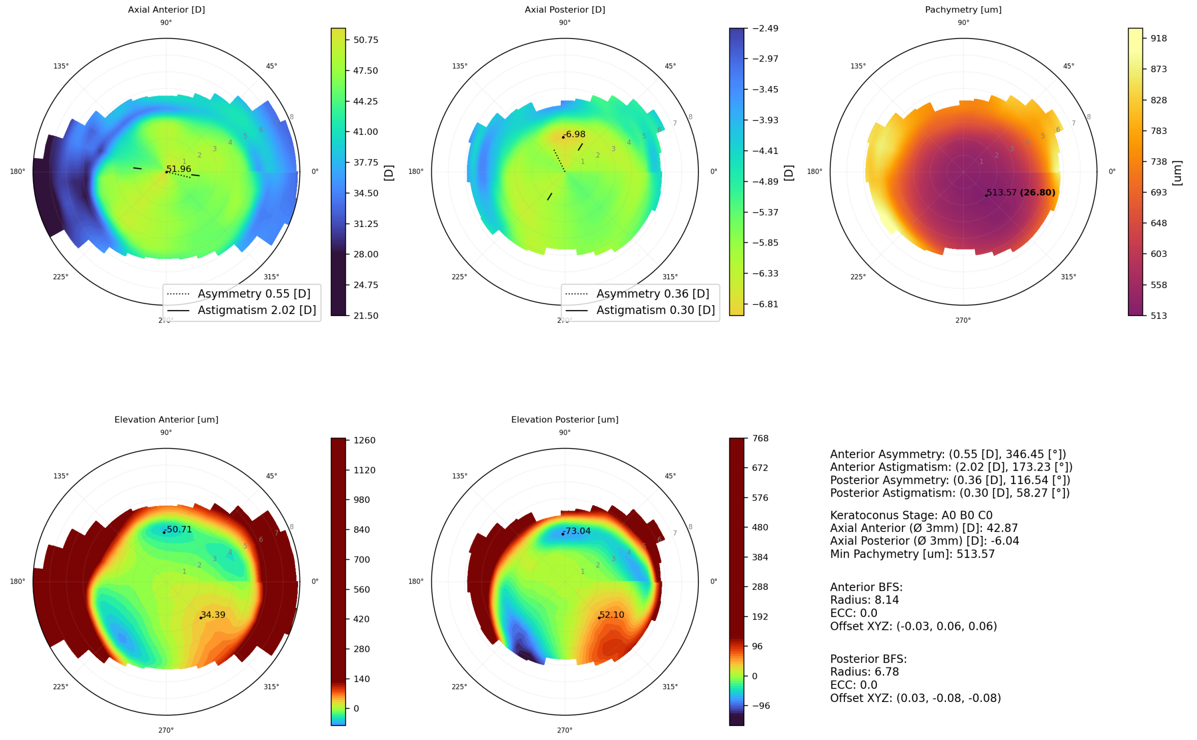

Figure S7. Scan labeled as "Other"

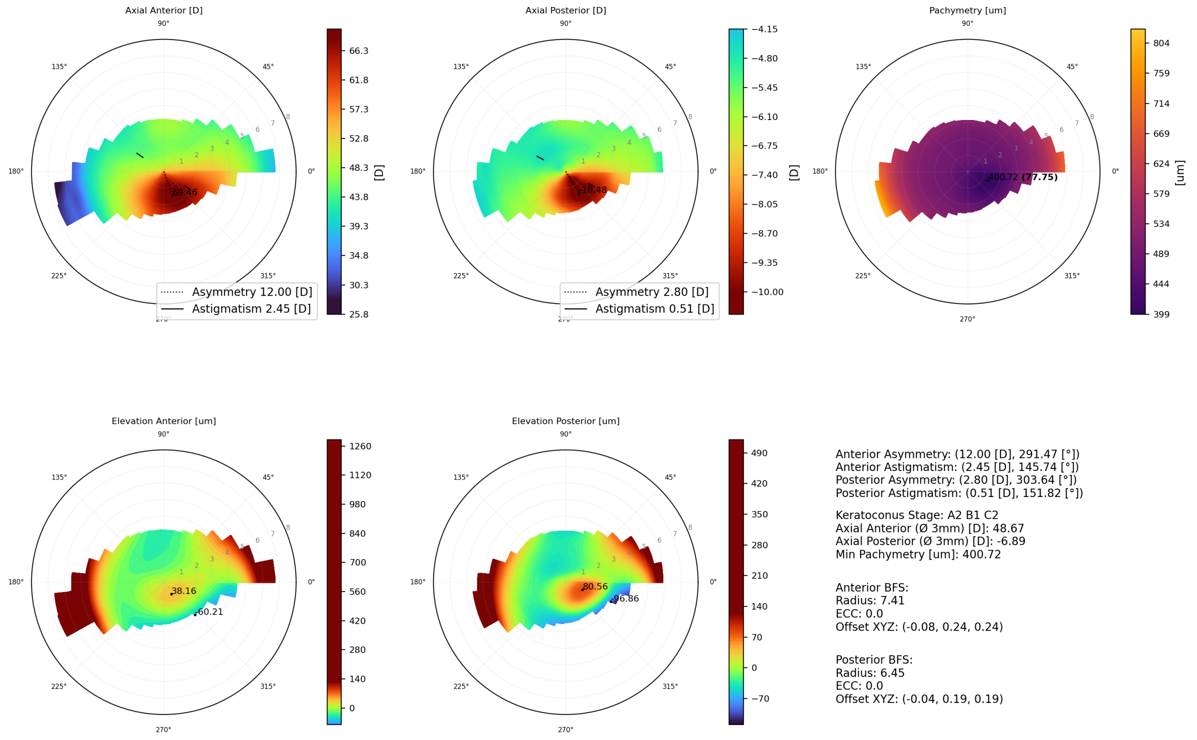

Figure S8. Scan labeled as "Not appreciable".

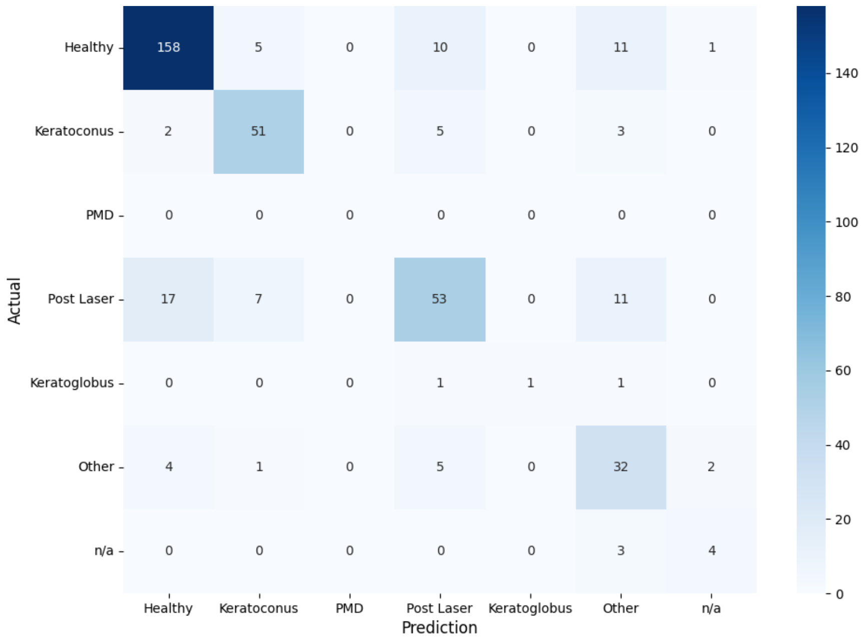

Figure S9. Confusion matrix of the validation cornea scans dataset. The *Prediction* axis shows the actual prediction from the *CorNeXt* model and the *Actual* axis shows the ground truth labels.

metric	balanced average	*Healthy*	*Keratoconus*	*Post Laser*	*Keratoglobus*	*Other*	*N/A*
accuracy	88.50%	87.11%	94.07%	85.05%	99.48%	89.69%	98.20%
F1 score	77.41	86.34	81.60	65.84	50.00	62.26	57.14
sensitivity	77.32%	85.41%	83.61%	60.23%	33.33%	75.00%	57.14%
specificity	96.22%	88.67%	96.02%	93.33%	100.00%	91.57%	99.21%
AUROC	0.9326	0.9565	0.9648	0.8826	0.9628	0.9275	0.9629

Table S1. Evaluation results of the final model on the validation cornea scans dataset. For the class *PMD* there are no samples in the validation dataset, so the class is omitted.

metric	*healthy* vs. *abnormal*
accuracy	87.11%
F1 score	88.03
sensitivity	90.64%
specificity	83.24%
AUROC	0.9553

Table S2. Evaluation results of the final model on the test cornea scans dataset when distinguishing only between *healthy* and *abnormal* cornea scans. *Post laser* is treated as *abnormal* in this case.
